# Bilateral adaptive deep brain stimulation is effective in Parkinson's disease

**DOI:** 10.1136/jnnp-2015-310972

**Published:** 2015-09-30

**Authors:** Simon Little, Martijn Beudel, Ludvic Zrinzo, Thomas Foltynie, Patricia Limousin, Marwan Hariz, Spencer Neal, Binith Cheeran, Hayriye Cagnan, James Gratwicke, Tipu Z Aziz, Alex Pogosyan, Peter Brown

**Affiliations:** 1 Nuffield Department of Clinical Neurosciences, John Radcliffe Hospital, University of Oxford, Oxford, UK; 2 Department of Neurology, University Medical Centre Groningen, University of Groningen, Groningen, The Netherlands; 3 Unit of Functional Neurosurgery, Sobell Department of Motor Neuroscience & Movement Disorders, UCL Institute of Neurology, London, UK; 4 Nuffield Department of Surgical Sciences, John Radcliffe Hospital, University of Oxford, Oxford, UK; 5 The Medical Research Council Brain Networks Dynamics Unit, University of Oxford, Oxford, UK

## Abstract

**Introduction & objectives:**

Adaptive deep brain stimulation (aDBS) uses feedback from brain signals to guide stimulation. A recent acute trial of unilateral aDBS showed that aDBS can lead to substantial improvements in contralateral hemibody Unified Parkinson’s Disease Rating Scale (UPDRS) motor scores and may be superior to conventional continuous DBS in Parkinson’s disease (PD). We test whether potential benefits are retained with bilateral aDBS and in the face of concurrent medication.

**Methods:**

We applied bilateral aDBS in 4 patients with PD undergoing DBS of the subthalamic nucleus. aDBS was delivered bilaterally with independent triggering of stimulation according to the amplitude of β activity at the corresponding electrode. Mean stimulation voltage was 3.0±0.1 volts. Motor assessments consisted of double-blinded video-taped motor UPDRS scores that included both limb and axial features.

**Results:**

UPDRS scores were 43% (p=0.04; Cohen’s d=1.62) better with aDBS than without stimulation. Motor improvement with aDBS occurred despite an average time on stimulation (ToS) of only 45%. Levodopa was well tolerated during aDBS and led to further reductions in ToS.

**Conclusion:**

Bilateral aDBS can improve both axial and limb symptoms and can track the need for stimulation across drug states.

## Introduction

Deep brain stimulation (DBS) is an established treatment for advanced Parkinson's disease (PD).^1^ However, its applicability is limited by costs, side effects and partial efficacy.^2^ That symptoms fluctuate based on cognitive/motor load, stress and medication status in PD is well established.^3^ Increasing evidence suggests that these fluctuations are associated with varying levels of subcortical β (13–30 Hz) oscillations, changes in the amplitude of which correlate with motor improvement in response to treatment.^4–7^ Building on a pioneering adaptive DBS (aDBS) study in the parkinsonian non-human primate,^8^ we recently successfully applied aDBS to patients with PD by triggering stimulation off the amplitude of β activity in the local field potential (LFP) recorded from the subthalamic nucleus (STN).^9^ This study showed that aDBS was more effective than continuous DBS (cDBS) despite <50% of the total time on stimulation (ToS). However, aDBS was applied unilaterally, with only 5 min stimulation and no assessment of axial symptoms nor control in different drug states.

Bilateral stimulation is essential if aDBS is to be progressed towards clinical applicability. However, this presents technical challenges. Although the subcortical β network is significantly coherent bilaterally,^10^ levels of amplitude co-modulation and phase synchronisation between STNs are low.^11^ This suggests that bilateral aDBS may require independent sensing and stimulating. We tested the efficacy of bilateral aDBS in PD, using independent bilateral sensing and stimulation, and in two patients we assessed how aDBS responded to levodopa administration.

## Patients and methods

We tested four male patients with advanced idiopathic PD undergoing DBS of the STN (table 1). All patients gave their informed written consent (approved by the local ethics committee). Patients underwent surgery in a two-stage procedure with bilateral quadripolar Medtronic (3389) electrode placement and stimulator implantation separated by 1 week, as previously described.^9^ ^12^ All testing was performed 2–6 days after electrode implantation.

**Table 1:** Clinical and stimulation details of patients

	Patient 1	Patient 2	Patient 3	Patient 4	Mean+SEM
Age (years)	66	41	52	54	53.3±5.1
Disease duration (years)	6	7	8	25	11.5±4.5
Preoperative UPDRS off-drugs	49	50	40	32	42.8±4.2
Preoperative UPDRS on-drugs	19	21	11	4	13.8±3.9
Surgery location	Oxford	London	London	London	
Initial symptom	Tremor	Bradykinesia	Tremor	Rigidity	
Primary DBS indication	Refractory tremor	ON-OFF fluctuations	Severe OFF states	OFF-related dystonia	
Preoperative drugs (mg/day)	Levodopa 1000 Pramipexole 3.15	Levodopa 625 Amantadine 100	Levodopa 500 Rotigotine 4	Levodopa 1600 Pramipexole 0.375 Tolcapone 800	
Online digital filter range (Hz)	20±3	21±3	25±3	20±6	21.5±0.8
Stimulation voltage (V, L/R)	3.4/3.4	3.4/3.3	2.5/2.4	3.0/3.0	3.0±0.1
aDBS ToS (%, L/R)	25/38	53/52	35/36	62/60	45.0±4.8

aDBS, adaptive deep brain stimulation; L, left; R, right; SEM, SE of the mean; ToS, time on stimulation; UPDRS, Unified Parkinson's Disease Rating Scale Part III (motor).

We recorded bipolar LFP activity from the STN electrode contacts after overnight withdrawal of anti-parkinsonian medication. Recordings were made using a band-pass (3–37 Hz) amplifier that has been previously described.^9^ ^13^ However, in this experiment, the amplifier and stimulator independently served both hemispheres and was miniaturised, fixed to the patient on their belt and connected to the base unit by a 10 m cable allowing assessment of ambulation and axial signs.

**Figure 1:**
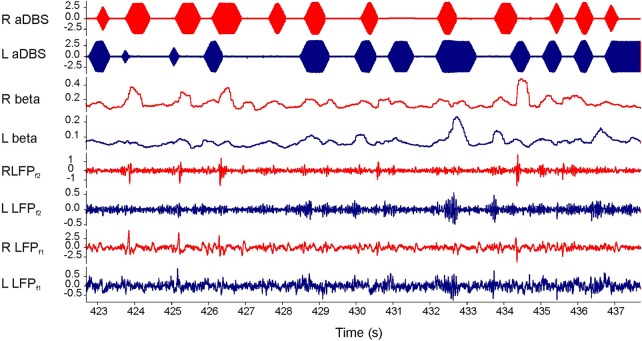
Screen shot of 15 s bilateral adaptive deep brain stimulation (aDBS) in patient 1. Bottom two traces are local field potentials (LFPs) after first stage analogue filtering (LFPf1; 3–37 Hz). Third and fourth traces from bottom show LFPs after second stage digital filtering around patient specific β peak (LFPf2; 20±3 Hz). The two traces above are the online readouts of the filtered β amplitude after rectification, smoothing and thresholding. The top two traces show bursts of ramped stimulation in response to β amplitude threshold crossing. Blue and red traces are from left and right electrodes, respectively. Note stimulation across the two sides is discontinuous and independent.

Bipolar recordings were made from the contact pair (0–2 or 1–3) exhibiting the greatest β amplitude determined for each hemisphere in the unstimulated and OFF-medication state. We then determined a single-centre frequency and bandwidth which encompassed both of these peaks and set this for our second stage of filtering (table 1). Online β amplitudes were obtained by rectifying and smoothing (400 ms moving average) the filtered LFP for each hemisphere. β Amplitude was then used to control triggering of stimulation via a user-defined threshold through a portable computer for each side independently.^9^ Stimulation was delivered by a dual version of our previously described stimulator.^9^ On triggering, stimulation was sustained until β amplitude fell below threshold (figure 1). Stimulation pulses were charge-balanced, monopolar and symmetrically biphasic (130 Hz, pulse width 100 µs, anodal pulse first then a 20 µs delay before the balancing cathodal pulse). An impedance of 0.5 kΩ was assumed for calculation of total electrical energy.

The stimulation voltage was determined during aDBS. The voltage was adjusted, along with the β amplitude trigger threshold, aiming for clinical benefit without paraesthesia, and a total ToS of approximately 50%.^9^ The contact selected for stimulation was that which lay between the contacts used for recording.^13^ Stimulation begun at 0.5 V and was increased by 0.1 V increments in order to reach a clinically effective voltage as determined by repeat assessments of rigidity and finger tapping by the attending (unblinded) neurologist. This voltage was then taken forward for formal testing and blinded assessment.

Voltage testing was performed separately, leading to two independent stimulation voltages (table 1). Patients were clinically assessed during aDBS and no stimulation. The order of experimental conditions was randomised. We compromised on a 5 min washout period between conditions so as to ensure the acceptability of the experimental session which then lasted about 1.5 h in two patients and 2.5 h in those two patients that went on to be stimulated during a levodopa treatment cycle. Stimulation (or no stimulation) was given for 15 min and clinical testing performed thereafter. In between experimental conditions, we tested the patients to confirm return to baseline before the next condition as judged through finger tapping.

Our primary outcome measure was the motor Unified Parkinson's Disease Rating Scale Part III (UPDRS III)^14^ with aDBS compared with no stimulation. Our secondary outcomes were power saving expressed as mean total electrical energy delivered with aDBS compared with similar, hypothetical, continuous stimulation and the correlation between β power and ToS. The patients were blinded by being kept unaware of the stimulation conditions throughout the experiment. In particular, the patients did not experience paraesthesia during aDBS. Although patients do sometimes experience paraesthesia during conventional DBS, the latter delivers more than twice the electrical energy used here. Bursts of stimulation during the aDBS practised here were short-lived, ramped in onset and offset and, on average, interspersed by periods of non-stimulation for 55% of the time (see table 1). UPDRS III assessments were also blinded by videoing them and having the videos rated (rigidity items excluded) by two external movement disorders specialists who were not involved in the experiment and were also unaware of the experimental conditions. There was strong concordance between the two raters with only an 11% average absolute difference between matched scores (p=0.85). UPDRS III scores were further divided into limb bradykinesia (items 8–17) and axial symptoms (arise from chair (18), gait (19), stability (21), posture (22) and overall bradykinesia (23)). Unblinded rigidity assessments were also reported separately for both experimental conditions.

Clinical data are described using means and SE of means, were normally distributed (Kolmogorov-Smirnov test, p>0.05) and analysed using two-sided t tests (t statistics with degrees of freedom shown along with p values). The first part of each recording during which the stimulation threshold was heuristically determined and any periods with marked electrical artefacts were excluded. We analysed the first (concatenated if necessary) section of 10 min artefact-free data after a stable threshold was obtained in each participant. Spectral analysis was conducted using Welch's method with a Hamming window (1 s). β Power and ToS were averaged over non-overlapping 10 s blocks and normalised to the average value of the first minute. To analyse the within-participant relation between mean β power per 10 s and total time on aDBS, between ToS per 10 s and total time on aDBS, and between ToS and β power per 10 s Spearman's correlations were estimated. Correlation coefficients were Fisher transformed, averaged and back-transformed. In patients 3 and 4, we performed additional, prolonged recordings of 45 and 60 min duration, respectively. At the beginning of these recordings, 100 mg levodopa was administered and the patients reported a transition from a clinical OFF to ON state which was confirmed by clinical examination of rigidity and bradykinesia. We performed change-point analysis to objectively identify when there was a simultaneous change in the ToS. Change-point analysis iteratively uses a combination of time-varying cumulative sum charts and bootstrapping to detect changes in time series.^15^ To avoid violation of the independent errors assumption, ToS was averaged over successive groups of three data points (where each data point was the average ToS over 10 s). Ten thousand bootstraps were performed in each test and only changes with probabilities of >99% shown. Significant changes in signal were depicted by means of different confidence limits. In one of the two patients (patient 4) a formal UPDRS III was performed, videoed and blindly rated during the levodopa challenge.

## Results

### Clinical effect

**Figure 2:**
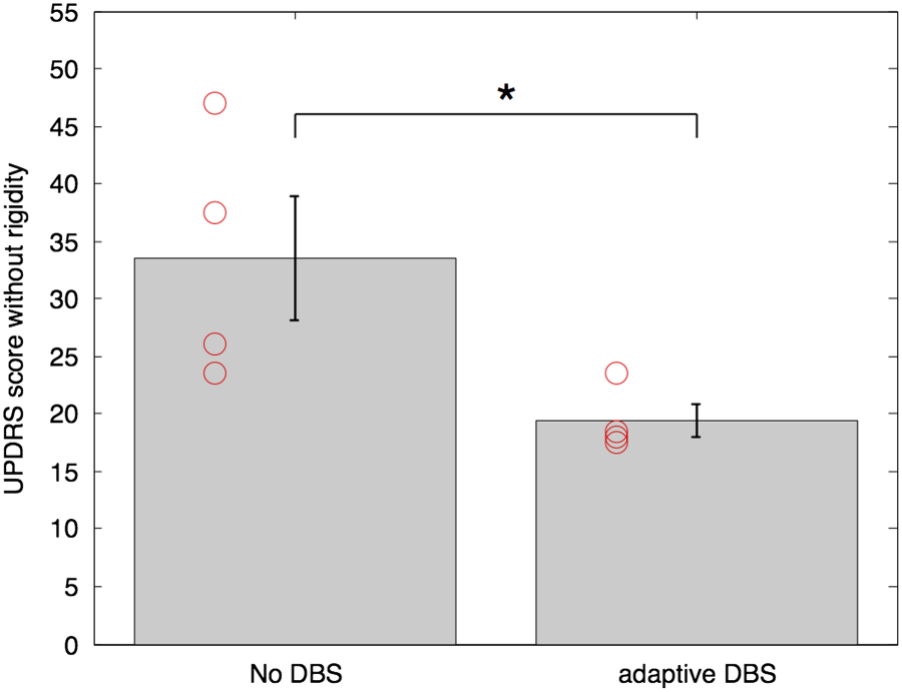
Group mean blinded UPDRS motor scores in the two experimental conditions and their SEs. *Indicates significance with p<0.05. Red circles depict individual data. DBS, deep brain stimulation; UPDRS, Unified Parkinson's Disease Rating Scale.

The mean UPDRS part III score OFF medication without stimulation in the days following surgery was 33.5±5.4 in the four patients. Individual and group data are illustrated in figure 2. There was a substantial improvement with aDBS in every participant (43% reduction: t_3_=3.24, p=0.04; Cohen's d=1.62). For limb bradykinesia and axial symptoms, the reductions were 37±10% and 39±5%, respectively. Remaining items, including tremor, speech, facial expression and freezing, improved by 55±12%. Unblinded rigidity scores were 9.5±1.5 in the condition without stimulation and 6.4±0.55 in the aDBS condition.

### Power savings

Total ToS for the whole stimulation period of aDBS was 45±4.8% averaged over the eight sides (4 subjects). Furthermore, LFP β amplitude and ToS tended to drop during the first 10 min. This was evidenced by the negative correlation between mean β power and total time on aDBS, and mean ToS and total time on aDBS across the eight sides. For β, the average Spearman's correlation coefficient was −0.23±0.08 (single sample t test, p=0.02). For ToS, the average Spearman's correlation coefficient was −0.20±0.15 (p=0.21). The mean total electrical energy delivered with aDBS was 223±31 µW. Conventional continuous stimulation at the same voltage would have delivered 491±44 µW.

### Response to levodopa

**Figure 3:**
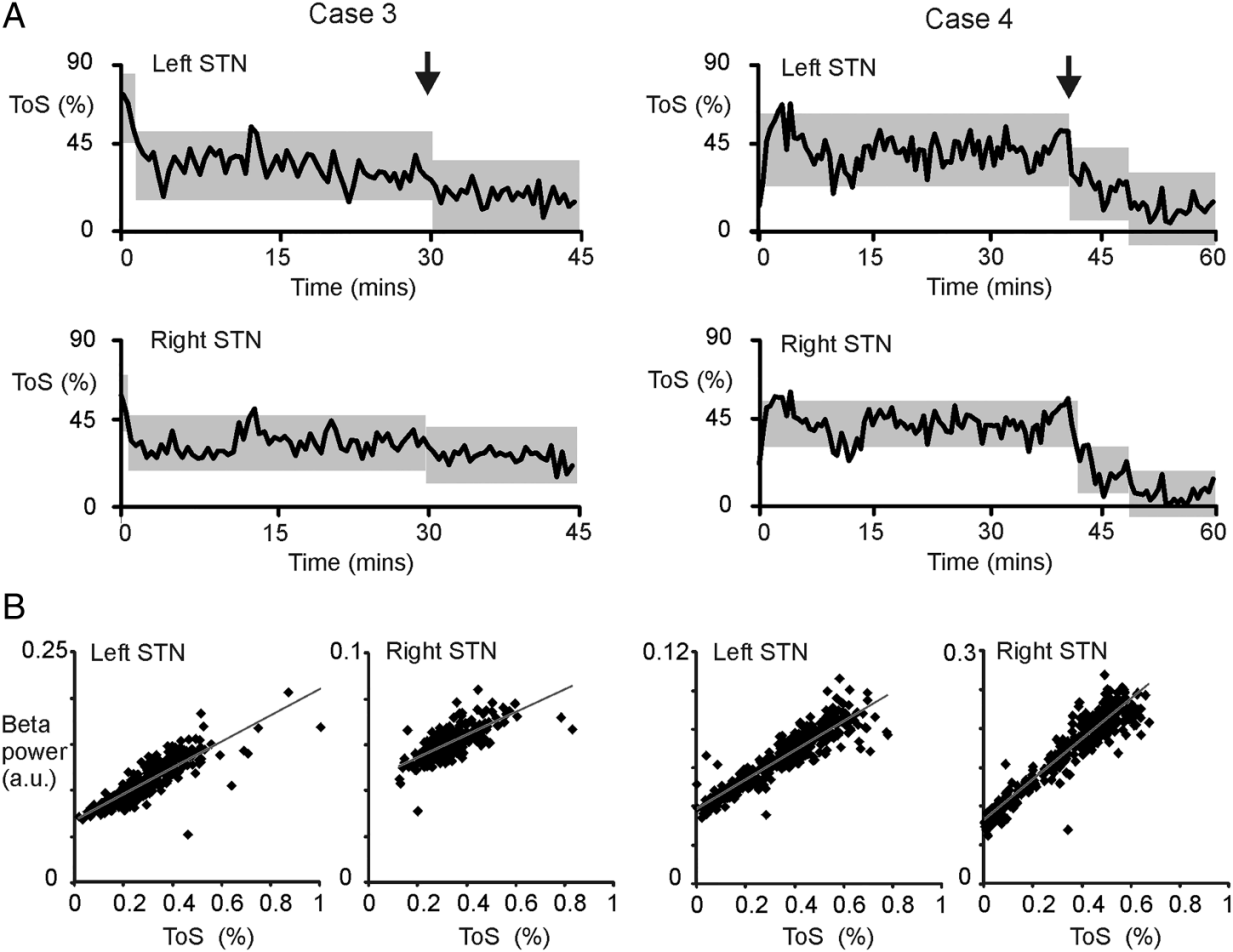
Per cent ToS during prolonged aDBS after levodopa administration at time 0 and its dependency on β power. (A) Grey blocks indicate the periods of stable ToS as identified by change-point analysis (p<0.01). The vertical extent of the blocks denotes the confidence limits of blocks between significant change points and these blocks are centred on the mean of the stable period. The vertical arrows denote when clinical improvement due to levodopa was first manifest. ToS dropped at the onset of the clinical effect of levodopa. Note that prior to this, ToS was stable for about 30 min or more, suggesting that the progressive drop in ToS at the very outset of aDBS plateaued with continued aDBS. (B) Scatter plots of β power and ToS per 10 s block. Grey line is the product of linear regression. aDBS, adaptive deep brain stimulation; STN, subthalamic nucleus; ToS, time on stimulation.

A prolonged recording following administration of levodopa medication in patient 3 resulted in a self-reported clinical effect 30 min after ingestion when the average ToS also significantly dropped (figure 3). In the prolonged recording in patient 4, a clinical effect was seen 40 min after levodopa. Again, the average ToS significantly dropped at this point (figure 3). Importantly, this drop in ToS occurred without deterioration in motor state, which instead improved from a blinded video-rated UPDRS III score of 17.5 with aDBS OFF drug, to 6.5 on aDBS and levodopa. The blinded video rating of items 1–8 of the Abnormal Involuntary Movement Scale was 1 out of 50. Note that on both sides of the two patients, change-point analysis of the ToS per 10 s was able to independently identify when patients turned ON after medication. Change point analysis of ToS (figure 3) also suggested that the progressive drop in β power and ToS seen at the very outset of aDBS plateaued with continued aDBS.

### Beta tracking

β Power showed a positive correlation with ToS in each participant in each hemisphere. The average Spearman's correlation coefficient was 0.62±0.12. In 7/8 hemispheres, this correlation was significant. In the recordings ON and OFF drugs in patients 3 and 4 (4 hemispheres) similar, even stronger, correlations were present. The average Spearman's correlation coefficient for these recordings was 0.884±0.147 (4 hemispheres).

## Discussion

We have previously shown that acute unilateral aDBS improves limb function more than unilateral cDBS.^9^ We demonstrate that bilateral aDBS using two autonomous sensing-and-stimulate systems is well tolerated and improves both axial and limb motor function. The mean effect size was a 43% reduction in blinded UPDRS scores relative to no stimulation and this was substantial with respect to the variability in the sample, as determined by Cohen's d. This large effect size of aDBS enabled statistically significant conclusions even though our sample was small. Moreover, we found that aDBS was appropriately modulated by levodopa administration, with a further reduction in stimulation, potentially preventing excessive combined therapy and dyskinesias. These findings suggest that bilateral aDBS may prove a safe, effective and highly efficient form of DBS in PD.

The scale of improvement with aDBS was striking, even though stimulation was only on for 45% of the time, averaged across the eight hemispheres. The latter is of critical importance as it impacts on both side effects and the life time of implanted battery systems. The 43% improvement with aDBS was similar to that seen with unilateral aDBS,^9^ and similar to that achieved with cDBS, when this has been similarly assessed through blinded rating of videos in other studies.^16–18^ Such reports tend to afford lower effect sizes than unblinded assessments, with blinded cDBS improvement estimates ranging between 25% and 38% for assessments of *chronic* bilateral stimulation with implanted systems^16–18^ and 31% with acute postoperative unilateral cDBS.^9^

Several experimental limitations may have biased against the detection of all but major effect sizes. Our sample size was small and stimulation regimes were tested only a few days after electrode implantation, when stun effects are present leading to increased electrode impedances and temporary improvement in baseline impairments.^19–21^ The presence of a stun effect in our sample was evidenced by the difference between the mean OFF-medication OFF-stimulation UPDRS score in the days following surgery (33.5±5.4, see Results section) and that recorded preoperatively (42.8±4.2, see table 1). We were also restricted to stimulating one of the middle two contacts of the implanted electrode on each side. Finally, there may have been incomplete washout between conditions. Although the randomised presentation of conditions should have ameliorated this in so far as incomplete washout should apply to both conditions, this cannot be relied on with a small sample, such as in this study, where order effects may still be influential. Furthermore, although the evidence suggests that the effects of conventional cDBS at least, are substantially, but not completely diminished after 5 min of washout, as used here,^22–24^ the time course of washout for axial symptoms is longer and the time course of the washout of aDBS is unknown. However, longer washout periods would have risked making the experiment unacceptably long for the immediate postoperative period. Nevertheless, these different factors were insufficient to obfuscate the large and significant effect size of aDBS.

The possibility that bilateral aDBS may be effective despite a mean ToS of 45% (with a comparable reduction in energy consumption) may prove to have major advantages in limiting stimulation side effects. Theoretical and empirical evidence suggests that conventional DBS can involve direct spread to local fibre tracts and interfere with physiological network activity and thereby compromise motor skills, including speech.^25^ Although current spread to fibre tracts will still occur with aDBS, overall side effects may potentially lessen as stimulation is more limited in time and delivered only when β network activity is pathologically synchronised.^26^ This potential for side effect reduction is underscored by the response of the stimulation system to levodopa administration. Stimulation on time was significantly reduced, in response to β suppression with levodopa.^4^

Overall, this study demonstrates that bilateral aDBS is well tolerated, improves total motor UPDRS III scores, and yet requires on average 45% of the standard ToS. The large effect size of aDBS enabled statistically robust conclusions even though our sample was small. Since β LFP activity is a relatively consistent feature of the STN in PD^2^ and consistent over long periods of time,^21^ ^27^ this approach has the potential to be realised clinically. However, much work remains to be done to explore and optimise the full range of parameters and algorithms that could be employed. Hereafter, the next step will be a head-to-head comparison of aDBS with standard continuous high-frequency DBS, with voltages and other stimulation parameters independently optimised for each stimulation regime in a large patient cohort.
